# Evaluating the Impact of the PoultryStar^®^Bro Probiotic on the Incidence of Bacterial Chondronecrosis with Osteomyelitis Using the Aerosol Transmission Challenge Model

**DOI:** 10.3390/microorganisms12081630

**Published:** 2024-08-09

**Authors:** Ruvindu Perera, Khawla Alharbi, Amer Hasan, Andi Asnayanti, Anh Do, Abdulkarim Shwani, Raj Murugesan, Shelby Ramirez, Michael Kidd, Adnan A. K. Alrubaye

**Affiliations:** 1 Cell and Molecular Biology Program, University of Arkansas, Fayetteville, AR 72701, USA; rperera@uark.edu (R.P.); ka030@uark.edu (K.A.); aasnayan@uark.edu (A.A.); ad086@uark.edu (A.D.); 2 Center of Excellence for Poultry Science, University of Arkansas, Fayetteville, AR 72701, USA; mkidd@uark.edu; 3 Department of Veterinary Public Health, College of Veterinary Medicine, University of Baghdad, Baghdad P.O. Box 1417, Iraq; amer.a@covm.uobaghdad.edu.iq; 4 Exotic and Emerging Avian Viral Disease Research Unit, Southeast Poultry Research Laboratory, U.S. National Poultry Research Center, Agricultural Research Service, USDA, Athens, GA 30605, USA; abdulkarim.shwani@usda.gov; 5 BIOMIN America Inc., 10801 Mastin Blvd Suite 100, Overland Park, KS 66210, USA; raj.murugesan@dsm.com (R.M.); shelby.ramirez@dsm.com (S.R.)

**Keywords:** chondronecrosis, osteomyelitis, lameness, probiotics

## Abstract

Bacterial chondronecrosis with osteomyelitis (BCO) lameness is a major welfare issue for broiler production worldwide affecting approximately 1.5% of broilers over 42 days old. Excessive body weight gain causes mechanical stress on long bones, leading to micro-fractures. This condition induces a bacterial infection of fractures, resulting in bone necrosis and eventual BCO lameness. Increasing gut integrity and supporting Calcium metabolism contribute to the optimal bone structure and subsequently reduce BCO lameness. Probiotics thus provide an excellent strategy for alleviating BCO due to the improvement of intestinal integrity and barrier function. Accordingly, the present study investigated the lameness reduction through the feed supplementation of a selected probiotic. Broiler chickens were assigned to three treatments, including a control litter group (FL), a PoultryStar^®^Bro probiotic fed group (BRO), and a control wire-flooring group (CW) designed to induce BCO lameness. The probiotic significantly decreased lameness by 46% compared to the control group (*p* < 0.05). The most predominant bacteria identified from the BCO lesions were *Staphylococcus cohnii* and *Staphylococcus lentus*. Moreover, significant increments of tight junction gene expression in jejunum and ileum, plus numerical improvements of body weight gain (BW; +360 g) and feed conversion ratio (FCR; −12 pts) were observed in BRO-supplemented birds.

## 1. Introduction

Bacterial chondronecrosis with osteomyelitis (BCO) lameness is a major animal welfare, food safety, and economic issue that affects the commercial broiler industry in the United States and many other countries worldwide [1,2,3]. BCO lameness results from the bacterial translocation and colonization of vulnerable joints (proximal heads of long bones) in heavy broilers [2]. Collagen-binding bacteria enter the bloodstream through paracellular translocation from the intestine and respiratory tracts, followed by hematogenous distribution into pre-existing osteochondrotic micro-fractures within the mechanically stressed and highly vascularized growth plates [2,4,5]. Blood capillaries within the growth plates may also be transected by the micro-fracture wounds, leading to focal hypoxia (ischemia) and cell death [2]. Necrosis and inflammation due to the persistent infection eventually manifest in macroscopic bone degeneration and visible lameness in broilers [5,6,7,8]. Based on the incremental increases in lameness attributable to BCO in the United States of America, it has been reported that BCO lameness infected at least 1.5% of broilers grown to processing weights post 42 d of age [2,9,10,11]. However, certain global episodic outbreaks report BCO incidences in flocks ranging from approximately 14% to 60% [12]. *Staphylococcus* spp. have been recognized as common and most important pathogens responsible for poultry skeletal infections [12,13]. Besides numerous species of staphylococci (*S. aureus*, *S. agnetis*, *S. cohnii*, *S. epidermidis*, *S. hyicus*, and *S. simulans*), other bacterial species such as *Enterococcus* spp. and *Escherichia coli* are frequently isolated from bone BCO lesions [12,14]. These results are affirmative with the commonly isolated bacteria from BCO lesions of lame birds from trials in our facility [4,8,15,16,17,18].

BCO lameness is entirely subclinical until the visual diagnosis of the broiler. Moreover, it is inherently intractable due to the animal’s short productive life cycle as well as the current broiler production model. Therefore, research into effective preventive treatment has become imperative for the industry to maintain high levels of productivity and animal welfare. Tight junction (TJ) protein complexes, which make up and maintain the epithelial integrity of the gut, have been shown to be critical in regulating intestinal permeability [17,19]. Compromised TJ proteins in chickens lead to increased intestinal permeability, which is frequently connected with poor health and performance, bacterial translocation, and immunocompromising, leading to infections like coccidiosis and others associated with BCO lameness [11,20,21,22]. Probiotic supplements have been known to benefit broiler growth performance by improving intestinal absorption, promoting a healthy balance of microorganisms in the digestive tract, strengthening intestinal barrier integrity, and optimizing metabolism while minimizing the susceptibility to pathogen invasion and intestinal inflammation [3,23,24,25].

The creation of a BCO-stimulating environment for the birds is crucial to test the effectiveness of mitigatory treatments. Wire-flooring models have been developed to impose both mechanical and physiological stress triggering BCO [9,11,26,27] as well as being a method to naturally spread the bacterial infection to other birds housed in the same facility. While this induction model is highly effective, it is also aggressive and can potentially reduce research translatability. Currently, our research group employs the aerosol transmission model, a validated and effective novel approach that mimics horizontal disease transmission in an industrial setting, to induce experimental lameness [4,28]. This approach utilizes seeder birds reared on two wire-flooring pens that can incubate the BCO etiological agents and spread them to the remaining birds reared on litter flooring in the same house via ventilation airflow. Shortly, two wire-flooring pens are placed on the left and right sides at the north (up-wind) end of the chicken house, followed by empty spaces as buffers. Two rows of litter flooring pens (for treatment groups and controls) are assembled downstream (downwind) on the left and right sides of the house, following the wire-flooring pens (BCO source group). Exhaust fans at the south end, along with automatic inlet ventilations on the sides direct the air flow from north to south (up- to downwind). The birds reared on wire-floor develop severe BCO due to exacerbated mechanical stress by walking on wire, and the air circulation facilitates the pathogen transmission to the rest of the house (downwind) via aerosol. Therefore, the wire floor-reared birds act as the source of natural BCO infection. This mimics the spread of disease in an industrial setting. A schematic of the model and further details were recently published [15]. Aerosolized bacteria can infect birds via the respiratory route. Additionally, the bacteria landing on the water nipples and feed can be ingested while feeding and drinking. Moreover, bacteria landing on the broilers’ feathers may be ingested while preening. Using this induction model, we focused on the identification of the bacterial causative agents of BCO lameness (to enhance the current understanding of the pathogens associated with BCO) and the testing of commercial probiotics, prebiotics, and synbiotics to reduce the incidence of BCO lameness in broilers [6,28,29]. In a previous trial, we observed a 20–25% reduction in incidences of lameness through the application of probiotics and selected feed supplements [6]. The decrement in lameness cases has been achieved by improving gut integrity via strengthening tight junctions and enhancing the bactericidal activity of peripheral blood monocytes [6]. To expand our study, the commercially available synbiotic PoultryStar^®^Bro was evaluated as a dietary supplement. This is a well-defined, poultry-specific, multi-species synbiotic which contains a blend of three carefully selected probiotic microorganisms (*Enterococcus faecium*, *Bifidobacterium animalis*, and *Pediococcus acidilactici*) and a prebiotic fructo-oligosaccharide. Previous work has been published with PoultryStar^®^ in wire-floor models for BCO [11,30], but this study is unique for using the aerosolized transmission model.

## 2. Materials and Methods 

### 2.1. Experimental Design

On d1, 480 one-day old Cobb 500 (Cobb-Vantress, Inc., Siloam Springs, AR, USA) male chicks were placed in 5 × 10 ft. pens on either suspended wire flooring [2,11] or wood-shaving litter at an initial density of 60 chicks per pen [6]. In accordance with the aerosol transmission model, two wire-flooring pens were placed side by side near the cooling pads at the front-end of the house. These 2 pens served as the source of BCO infection (CW group). The other fresh litter-flooring pens were placed downwind behind the wire-flooring pens, in two rows, extending towards the exhaust fans. The fresh litter-flooring pens contained the birds of the other 2 treatments: control group (FL) and the probiotic-fed group (BRO). While the CW group had only two pens, the FL and BRO groups had three pens/group in a random array (Table 1). 

On d14, all pens were culled to 55 birds to maintain optimum stocking density. All pens received standard commercial chick starter feed (crumbles) from d1 to d34 and broiler finisher (pellets) from d35 to d56, which were prepared at the University of Arkansas feed mill. Birds of the CW and FL groups received a basal diet, while the BRO group received the PoultryStar^®^Bro probiotic as a dietary supplement (500 g per MT of diet) added to the same basal diet. Feed consumption was determined per pen from placement to 56 d. In each pen, two feeders were placed on one side, and water lines with nipple drinkers on the opposite side, to encourage natural bird movement. All water pipelines were disinfected with 10% bleach followed by fresh water flushing prior to bird placement. All birds had access to feed and clean water ad libitum throughout the trial. 

### 2.2. Environmental Conditions

The research barn was equipped with computer controllers regulating temperature, photoperiod, and ventilation. Tunnel ventilation and evaporative cooling pads were automatically activated at preset ambient temperatures. The photoperiod was set at 23L:1D for the duration of the trial. Thermoneutral temperature targets were as follows: 32 °C from d1 to d3; 31 °C from d4 to d6; 29.5 °C from d7 to d10; 26.5 °C from d11 to d14; and 24 °C thereafter.

### 2.3. Evaluation of Lameness

Starting from d22, all birds in both types of treatment pens were gently prompted to move and walk for lameness assessment. Birds that were unwilling to walk, walked with difficulty, or were unable to walk were diagnosed as “clinically lame” and humanely euthanized. All birds that were found dead or to have developed clinical lameness were recorded by date and pen number and necropsied for BCO lameness categorization [2]. Cumulative lameness per pen, via necropsy, was also conducted beginning on d22. Each dead and/or necropsied bird was assigned to at least one of the following categories: N = Femur head and proximal tibia appear entirely normal; Cull = Runts and individuals that failed to thrive or appeared to be clinically ill; DUR = Death due to Unknown Reasons; SDS = Sudden Death Syndrome (Flipover, Heart Attacks); KB = Kinky Back (Spondylolisthesis); TW = Twisted Leg or Slipped Tendon (perosis); TD = Tibial Dyschondroplasia; Lame; UNK = Lameness for undetermined reasons; FHS = Proximal Femoral Head Separation (epiphyseolysis); FHT = Proximal Femoral Head Transitional degeneration; FHN = Proximal Femoral Head Necrosis (bacterial chondronecrosis with osteomyelitis, BCO); THN = Proximal Tibial Head Necrosis; THNC = Proximal Tibial Head Necrosis Caseous; THNS = Proximal Tibial Head Necrosis Severe; Total Lame = FHS + FHT + FHN + THN +KB. 

### 2.4. Bacterial Species Identification 

On d56, two lame birds per pen were randomly chosen for bacterial isolation from proximal tibial and femoral head lesions using cotton swabs. Samples were plated separately in CHROMagar^TM^ Orientation and CHROMagar^TM^ *Staphylococcus* (DRG International, Springfield, NJ, USA) media and incubated overnight at 37 °C for bacterial CFU (Colony Forming Units) enumeration. The total number of colonies and morphologies were recorded, followed by re-streaking isolated colonies on fresh media plates. For DNA extraction (using an in-house NaOH extraction method), one to two colonies were picked from the aforementioned cultures (using sterilized tooth-picks) and suspended in 90 μL of sterile water in wells of a PCR plate. Next, 10 μL of 1 M NaOH was added and incubated for 10 min at room temperature. A PCR amplification of DNA was then performed, using ribosomal RNA (rRNA) as a biomarker for the identification of bacterial species. Conventional PCR was used for the amplification of the rDNA 16S V1-V5 region. In detail, 50 μL total PCR mixture was prepared from 25 μL of Phusion^®^ High-Fidelity PCR Master Mix (New England Biolabs^®^ Inc., Ipswich, MA, USA), 0.5 μM of each forward (5′-AGAGTTTGATCCTGGCTCAG-3′) and reverse (5′-GTGCGGGCCCCCGTCAATTC-3′) primer, 1.5 μL of dimethyl sulphoxide (DMSO), 2 μL of DNA samples, and 20.5 μL of nuclease-free water. The following settings were used by the thermal cycler (Bio-Rad T100^TM^, Hercules, CA, USA) for PCR: 98 °C for 30 s, 98 °C for 10 s, 71 °C for 30 s, 72 °C for 30 s (35 cycles), 72 °C for 3 min, followed by 4 °C infinite hold. The presence of the targeted DNA fragments was verified via 1% agarose gel electrophoresis (0.5× TBE buffer at 60–70 V). A Diffinity Rapid Tip (Diffinity Genomics, New York, NY, USA) kit was used for the PCR product purification. In total, 40–60 ng/µL of the purified DNA along with 2–10 pmol/µL primer were shipped for sequencing to Eurofins Genomics, Louisville, KY, USA. Sequencing results were analyzed using DNASTAR Lasergene software v17.3 (DNAStar, Inc., Madison, WI, USA) and compared with the National Center for Biotechnology Information (NCBI) (https://www.ncbi.nlm.nih.gov/ accessed on 10 December 2020) and the Ribosomal Database Project (RDP) (http://rdp.cme.msu.edu/ accessed on 10 December 2020) databases. The species was determined with ≥95% sequence similar identity with the databases.

### 2.5. Tight Junction Gene Expression

Five birds per treatment, chosen at random were subjected to cervical dislocation and necropsy for jejunum and ileum tissues on d56 of the experiment. The mesentery and connective tissues were removed, and segments of mid-jejunum and mid-ileum were extracted. Then, they were cleansed with ice-cold phosphate-buffered saline (pH 7.4), submerged in RNAlater (Sigma Aldrich, St. Louis, MO, USA) and stored at −20 °C for further analysis. The expression of 3 TJ genes, Occludin (OCLN), Claudin-2 (CLDN-2), and Zonula occludens-1 (ZO-1), was analyzed. RNA extraction was performed using an acid guanidinium thiocyanate-phenol-chloroform extraction method [31]. Complementary DNA (cDNA) was synthesized based on a modified version of the protocol proposed by Gubler and Hoffman (1983) [32]. Finally, the relative expressions of the TJ genes were evaluated via qPCR (of the cDNA synthesized from mRNA transcripts).

### 2.6. Statistical Analysis

Statistical differences were evaluated for the FL and BRO treatment groups, as well as the CW group which was an aerosol source of naturally occurring BCO pathogens. The incidence of FSH, FHT, FHN, THN, and TD were calculated as the sum of the right and left bones over the number of placed birds multiplied by 2 bones sampled. A projected feed conversion ratio (FCR) was calculated with the formula:

$$\left[\left\{\frac{Pen\;feed\;intake}{\left(Pen\;weight\;gain\;+\;mortality\;weight\right)}\right\}/Bird\;days\right]\times \left(56\;days\times 60\;birds\right)$$

due to the high mortality observed in the study, to reflect feed conversion more accurately. Continuous data were analyzed using the GLIMMIX procedure of SAS 9.4 (Cary, NC, USA) using a Gaussian distribution whereas frequency data were analyzed using a binomial distribution and back-transformed using ILINK function. Data were reported as least square means (LSMeans) and the standard error of the mean (SEM) was pooled. Statistical differences were reported at *p* < 0.05. The relative fold gene expressions of the TJ genes were obtained by using the ∆∆Ct (Delta-Delta-Ct) method in Ms. Excel 365 software v16.0 (Microsoft, Redmond, WA, USA), with TATA box binding (TBP) protein gene as the reference gene.

## 3. Results

### 3.1. Daily Cumulative Lameness

**Figure 1:**
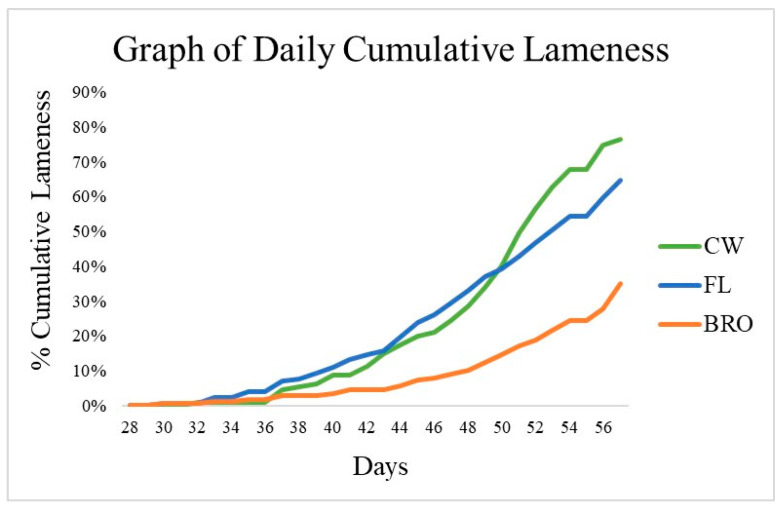
Cumulative percentage of lameness for birds raised on wire-floor (CW), birds raised on fresh litter (FL), and birds raised on fresh litter fed PoultryStar^®^Bro (BRO) over 56 days. From d40 to d56, BRO birds had significantly (*p* < 0.05) less lameness than FL birds.

Beginning on d22, birds were assessed for lameness by evaluating their resistance to walking. The first case of lameness was observed on d30. Between treatment groups, there was no observable difference in the age of onset of lameness. There was no treatment difference (*p* > 0.05) in daily cumulative lameness between FL and BRO birds from d15 to 39; however, from d40 to 56, birds fed BRO had reduced (*p* < 0.05) lameness compared with FL birds (Figure 1).

The overall cumulative lameness of FL birds was 64.7%, which was numerically less than the reference CW birds (76.7%). However, this also indicated that the CW birds had successfully spread the aerosol infection “downwind” to birds raised on litter. Birds fed BRO had reduced (*p* < 0.05) cumulative lameness incidence by 46% compared with FL birds. The total mortality in the CW birds was 97.4%, which included those removed for lameness. Birds fed BRO had a maximum total mortality of 52.0%, which is a significant reduction (*p* < 0.05) of 40% when compared with the total mortality of FL birds (87.3%).

**Figure 2:**
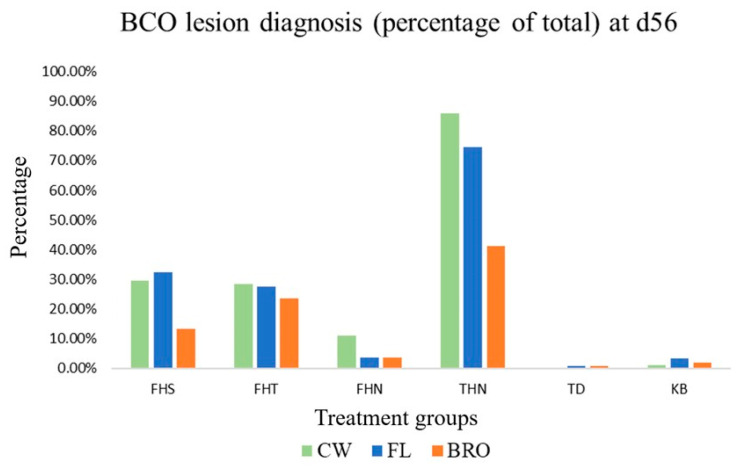
Lesion diagnosis (FHS—Femoral Head Separation; FHT—Femoral Head Transitional; FHN—Femoral Head Necrosis; THN—Tibial Head Necrosis; TD—Tibial Dyschondroplasia) for lame birds and the percentage of Kinky Back (KB) birds raised on wire-flooring (CW), birds raised on fresh litter fed control diet (FL), and birds raised on fresh litter fed PoultryStar^®^Bro (BRO) on d56. No significant differences (*p* < 0.05) were observed between treatments for the incidence of BCO lesions.

Non-lame mortalities were primarily due to unknown reasons or caused by Sudden Death Syndrome. There were no differences (*p* > 0.05) between FL and BRO birds for the incidence of FHT, FHN, TD, or KB. Birds fed BRO had a reduced (*p* < 0.05) incidence of FHS and THN compared with FL birds (Figure 2). In overall, the majority of lameness was due to FHS, FHT, or THN regardless of treatment group.

### 3.2. Bacterial Species Diagnosis from BCO Lesions

Eight different bacterial species isolated from BCO lesions of the femoral and tibial heads were identified and listed in Table 2. Considering the total population of subjects, the predominant bacterial species present in the BCO lesions was *Staphylococcus cohnii* (67.44%), followed by *Staphylococcus lentus* (13.95%). The remaining species were less prominent. 

### 3.3. Feed Conversion Ratio (FCR) and Body Weight (BW) Gain

The primary objective of this study was not the evaluation of bird growth performance and FCR. But body weight (BW) gain and projected FCR were calculated, as depicted in Table 3.

Birds fed BRO had numerical improvements in BW gain (+360 g) and FCR (−12 pts); however, these were not statistically significant. The numerical improvement observed was greater than birds fed *Bacillus subtilis* (+30 g) compared with controls from a previous study regardless of flooring type; however, FCR was not reported in the cited study [30]. Additionally, the current study observed numerical improvements in BW, which were greater than the BW of birds fed *Bacillus subtilis* (+130 g) and *Bacillus licheniformis* (+50 g); however, FCR was not reported in this cited study either [33]. Yet, it is important to note that not all probiotics work similarly, i.e., they differ based on their mode of action. While most *Bacillus* products are transient and function in the lumen, PoultryStar^®^ products colonize the gut [34] for competitive exclusion and improvements in intestinal integrity.

### 3.4. Tight Junction Gene Expression

**Figure 3:**
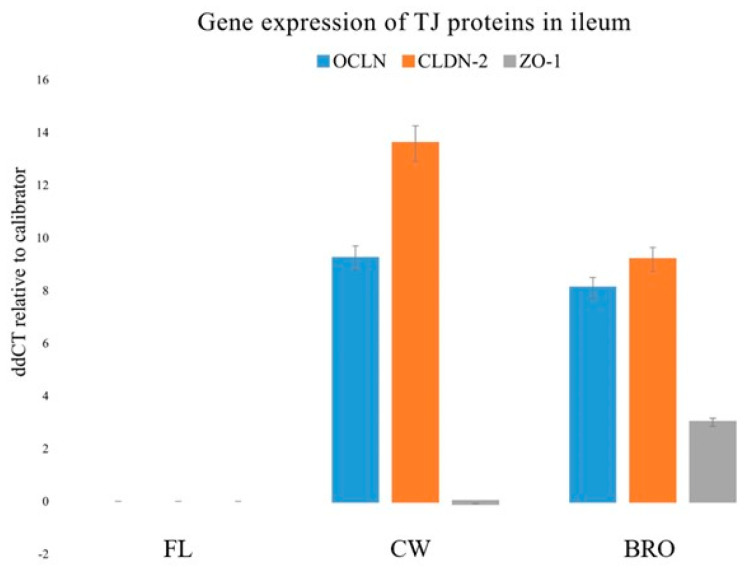
Relative gene expressions (using the delta-delta Threshold Cycle (ddCT or 2^−ΔΔCt^) method) of OCLN (Occludin), CLDN-2 (Claudin-2), and ZO-1 (Zonula occludens-1) genes in the ileum of birds of the control (fresh litter; FL), BCO source (control wire-flooring; CW), and PoultryStar^®^Bro probiotic-fed (BRO) groups using the fresh litter group as the reference.

The present study examined the gene expressions of three tight junction proteins in the jejunum and ileum, namely, Occludin (OCLN), Claudin-2 (CLDN-2), and Zonula occludens-1 (ZO-1). Bacterial infection dramatically decreased OCLN, CLDN-2, and ZO-1 gene expression in the ileum, but probiotic administration significantly boosted OCLN, ZO1, and CLDN -2 mRNA expression (Figure 3).

**Figure 4:**
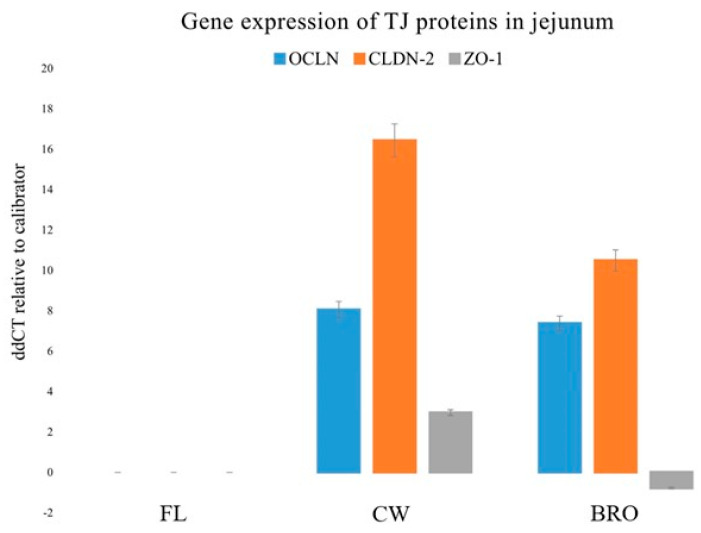
Relative gene expressions (using the delta-delta Threshold Cycle (ddCT or 2^−ΔΔCt^) method) of OCLN (Occludin), CLDN-2 (Claudin-2), and ZO-1 (Zonula occludens-1) genes in the jejunum of birds of the control (fresh litter; FL), BCO source (control wire-flooring; CW), and PoultryStar^®^Bro probiotic-fed (BRO) groups using the fresh litter group as the reference.

Likewise, bacterial infection significantly decreased OCLN, ZO1, and CLDN-2 gene expression in the jejunum, but probiotic supplementation boosted OCLN gene expression (Figure 4).

The increment of the expression of the above transcripts for important tight junction proteins indicates the strengthening effect of BRO on the gastrointestinal tight junctions, and therefore the barrier integrity as a whole.

## 4. Discussion

The present study included three treatment groups consisting of a control litter group (FL), PoultryStar^®^Bro (BRO) probiotic-fed group, and a control wire-flooring group (CW) designed to induce BCO lameness in the birds. This study aspires to provide valuable information regarding the use of commercial probiotic supplements to optimize bird health and well-being, as well as minimizing incidences of lameness that are currently impacting the industry. The onset of lameness of the present study initiated on d30, which is consistent with the reported onset of BCO in commercial conditions between 4 and 6 weeks of age [35]. The highest onset of final percentage cumulative lameness in a previous experiment [11] was 68% on wire-flooring, but the litter-flooring control group only experienced 12% lameness when the models were used separately. Moreover, in another study [6], when a wire-flooring model was used alone, the control group (not treated with probiotics) experienced a final cumulative lameness of 66%. However, with the model used in the present study, not only did the wire-flooring-raised birds (the source of infection) develop higher final cumulative lameness (76.7%) than that of the previous studies, but they also induced 64.7% lameness in the litter-flooring-raised control birds. These statistics affirm and further validate the effectiveness of the aerosol transmission model to induce lameness in birds raised on litter flooring, the incidence rate of which rivals that observed in wire-flooring pens. Therefore, the aerosol transmission model is evidently successful as a lameness stimulating model. The 46% decrease in lameness incidence observed in the BRO group is also consistent with previously published data, where probiotics reduced lameness between 44 and 71% compared to the respective control group [11]. The probiotic blend used in the experiments discussed in Wideman et al. (2012) [11] was similar to that in BRO except for the inclusion of *Lactobacillus reuteri.* Other probiotic blends, such as the bacterial spore-former *Bacillus subtilis*, have also been evaluated to minimize BCO lameness [30]. This *B. subtilis* reduced the incidence of BCO by 41%, which was lower than that observed in the current study (46%). Interestingly, in the same study, when enrofloxacin was used at a therapeutic dose from d35 to d54, lameness was decreased by approximately 50%; however, when enrofloxacin was removed for the remainder of the trial, the overall cumulative lameness was reduced by 34% compared with the control. This demonstrates that the therapeutic usage of an antibiotic is effective at lowering lameness during the time of administration; even so, the continuous use of a probiotic as a preventative measure is more effective in reducing overall BCO lameness. Furthermore, within the wire-flooring model, a lameness reduction of 20% was recorded [11] between the control and probiotic-administered diets when PoultryStar^®^ was used. These findings suggest the excellent capability of probiotic supplements to lower lameness in both BCO-stimulating (wire-flooring) and normal litter environments, as indicated by the 46% lameness decrement observed in the present study. 

Numerous bacterial species are associated with BCO lesions, including *Staphylococcus* spp., *Escherichia coli*, *Salmonella*, and *Enterococcus* [27]. They can originate from parent stock (e.g., vertical transmission), the respiratory inhalation of aerosolized materials, or via the gastrointestinal tract after the ingestion of contaminated feed or water or the intake of feather dust during preening [9]. In a more recent study, *E. cecorum*, *E. coli*, and *S. aureus* were the most predominant bacterial species found in BCO lesions [36] and are involved with the pathogenicity of BCO. In contrast, air sampling in the same commercial broiler farms of Arkansas resulted in a majority of *S. cohnii* (∼95%) along with 3–4% *Staphylococcus lentus* and 1–2% *E. coli* being discovered. Moreover, another study [28], which used the wire-flooring model in combination with the *S. agnetis* challenge in drinking water, found that the lame birds had a predominance of other *Staphylococcus* species (*S. saprophyticus*, *S. pseudintermedius*, *S. haemolyticus*, and *S. xylosus*) in BCO lesions, in addition to the expected *S. agnetis*. This variation from study to study (the present study included) further indicates that BCO has complex pathogenicity involving many causative agents, environmental conditions, and predisposing factors that warrant further research for better understanding. Despite eight species of bacteria being isolated from BCO lesions in this study (Table 2), whether they all contribute to the pathogenicity of BCO warrants further research.

Probiotics are generally live bacteria that influence the host through the competitive exclusion of microbiota, enhancing the intestinal barrier function, improving bone tissue integrity, and modulating the immune responses [37,38]. It is important to note that not all probiotics or synbiotics are the same, and depending on the strain or form used, results may vary. Two bacterial spore-formers, *Bacillus subtilis* and *Bacillus licheniformis*, were used previously by Alrubaye et al. (2020) [6] and resulted in a 20% and 25% reduction in lameness compared to their respective controls. Only *B. licheniformis* was able to increase transepithelial resistance (a measure of intestinal integrity) compared to the respective control treatment. In the above study, source birds were inoculated with *Staphylococcus agnetis*, while the remaining birds were infected through aerosolized agents. The PoultryStar^®^ product’s ability to competitively exclude intestinal pathogenic bacteria is well depicted in a recent study [34] where PoultryStar^®^ achieved significant reduction in *Salmonella* Enteritidis (SE) in the cecal content of broilers, compared to control. Additionally, the relative abundance of IL-10, IL-1, TLR-4, and IFNγ mRNA was significantly reduced in the probiotic-supplemented group, further depicting the normal status of cecal tonsils, because of SE load reduction.

One strategy to reduce BCO lameness is to enhance gastrointestinal defense, which comprises several barriers such as the mucus layer, epithelial cells connected via junctional complex proteins, and immune cells [39,40]. The gastrointestinal epithelium is a crucial physical barrier of the innate immune system that inhibits luminal antigens or bacteria from crossing the mucosa to eventually reach the blood stream. The strength of the gastrointestinal barrier system is dictated by tight junctions, which are intercellular adhesion complexes in epithelia and endothelia that control paracellular permeability. Probiotics are known to reduce bacterial translocation via barriers, thereby contributing to control BCO lameness [6,11,30]. The present study portrays the probiotic-mediated increment of gastrointestinal tight junction gene (OCLN, CLDN-2, and ZO-1) expression (Figure 3 and Figure 4) and the strengthening of the barrier as a whole. In similar studies, lactic acid bacteria (LAB) probiotics, have beneficially affected intestinal integrity and barrier function during times of heat stress [41,42]. Song et al., (2014) [43] fed birds a mixture of LAB in thermal neutral and heat stress conditions. Results indicated that irrespective of environmental temperature, the blend improved the villus height-to-crypt depth ratio, tight junction protein expression, and transepithelial resistance while reducing intestinal permeability.

Another means of minimization of lameness is the strengthening of the bone tissue by improving its structural integrity [44], and probiotics are known to regulate the development of bones and metabolism via the regulation of the host immune system through their metabolites [45]. Furthermore, a recent study [46] evaluated the ability of synbiotics to function as alternatives to antibiotics in heat-stressed broilers. It was observed that birds with a dietary supplementation of PoultryStar^®^ synbiotics exhibited resistance to heat shock, with significant improvements in skeletal health in comparison to both the antibiotic (bacitracin methylene disalicylate)-supplemented and basal diet-fed (control) groups. Another study [47] investigated the effects of the dietary supplementation of a commercial symbiotic (containing a prebiotic (fructo-oligosaccharides) and *Enterococcus faecium*, *Pediococcus acidilactici*, *Bifidobacterium animalis*, and *Lactobacillus reuteri*) on broiler bone health under daily cyclic heat stress. Overall, the symbiotic supplementation significantly increased the bone mineral density, bone mineral content, and bone area and improved the birds’ ability to stand for longer, while simultaneously decreasing lameness incidence in comparison to the basal diet.

In terms of augmenting host immune responses, *Clostridium butyricum* increased the antioxidant activity and decreased the Malondialdehyde content in the serum of broilers, thereby improving antioxidant capacity and, in turn, immunity. Furthermore, the above effects were amplified by administering the probiotic *Clostridium butyricum* synergistically with a prebiotic 1, 25-dihydroxyvitamin D3 [45]. Moreover, a dietary probiotic blend in both viable and inactivated forms [48] showed anti-inflammatory effects on cecal tonsils in broilers via the significant downregulation of inducible Nitric Oxide Synthase (iNOS) compared to control. Evidently, the potential of probiotics to minimize lameness by enhancing the intestinal barrier function and bone structural integrity, and modulating of immune responses is promising. Briefly, the present study successfully demonstrates the ability of a probiotic dietary supplement to significantly reduce lameness in chickens while strengthening their gastrointestinal barrier integrity. Furthermore, the prevalent bacterial species associated with BCO lameness are elucidated. The limitations of the present study include histopathological evaluations of BCO lesions as well as immunological analyses (e.g., immunohistochemistry, Western blot, and ELISA) of improvements of probiotic-induced response against pathogens. These limitations warrant further investigations to facilitate a wholistic understanding of the actions of PoultryStar^®^Bro in lameness prevention.

## 5. Conclusions

The probiotic PoultryStar^®^Bro led to 35% cumulative lameness with approximately 46% reduction in BCO incidences compared to the negative control group. Thereby, BRO significantly reduced BCO lameness incidence. Secondly, the control litter-flooring-raised birds developed almost similar levels of lameness (64.7%) as the wire-flooring-raised birds (76.7%). Thereby, the aerosol transmission model is highly successful in lameness induction.

## Figures and Tables

**Table 1:** Description of treatment groups.

Treatment	Description	Pens ^1^
CW	Diet 1 (Basal diet with no probiotics): wire-flooring pens	2
FL	Diet 1 (Basal diet with no probiotics): negative control group, litter flooring pens	3
BRO	Diet 2—PoultryStar^®^Bro (500 g/MT) added to the basal diet: litter flooring pens	3

CW; Wire-flooring group, FL; Fresh litter group, BRO; PoultryStar^®^Bro probiotic fed group; ^1^ Number of birds per pen (n) = 60 birds.

**Table 2:** Bacterial species identified on BCO lesion samples extracted from femoral and tibial heads of the long bones.

Bacterial Species	% Recovered from Femur	% Recovered from Tibia	Total % Recovered
*E. faecalis*	100	~	2.33
*E. cecorum*	100	~	2.33
*S. simulans*	100	~	4.65
*S. cohnii*	96.55	3.44	67.44
*S. agnetis*	~	100	2.33
*S. sciuri*	100	~	2.33
*Corynebacterium stationis*	100	~	4.65
*S. lentus*	66.667	33.30	13.95

The birds used for the above analysis were randomly chosen (two birds/pen) on the final day (d56) of the lameness trial. Bacteria were isolated from proximal tibial and femoral head lesion, grown on culture plates, and subjected to DNA extraction, followed by a PCR amplification of the rDNA 16S V1–V5 region. The amplified products were purified and sequenced for species identification.

**Table 3:** BW gain (d 0–56) and FCR for birds raised on fresh litter fed a control diet (FL) and birds raised on fresh litter fed PoultryStar^®^Bro (BRO) ^1^.

Treatment	BW Gain, kg	FCR, kg:kg
FL	3.55 ^a^	2.22 ^a^
BRO	3.91 ^a^	2.10 ^a^
SEM	0.69	0.08
*p*-value	0.735	0.370

^1^ SEM and *p*-value represent the standard error of the means and statistical significance of the difference, respectively. FCR was calculated based on projected performance with 60 birds per pen. Superscripted letter ^a^ indicates significant differences (within the same column of this table) at *p* < 0.05.

## Data Availability

Raw data supporting the conclusions of this manuscript will be available upon request.
